# The Research Landscape of Multiple Endocrine Neoplasia Type 1 (2000–2021): A Bibliometric Analysis

**DOI:** 10.3389/fmed.2022.832662

**Published:** 2022-04-08

**Authors:** Chenzhe Feng, Haolin Chen, Leyi Huang, Yeqian Feng, Shi Chang

**Affiliations:** ^1^Department of Oncology, The Second Xiangya Hospital of Central South University, Changsha, China; ^2^Department of General Surgery, Xiangya Hospital of Central South University, Changsha, China; ^3^Department of Mathematics, University of California, Davis, Davis, CA, United States; ^4^Xiangya School of Medicine, Central South University, Changsha, China; ^5^Hunan Provincial Engineering Research Center for Thyroid and Related Diseases Treatment Technology, Changsha, China; ^6^Clinical Research Center for Thyroid Disease in Hunan Province, Changsha, China

**Keywords:** Multiple Endocrine Neoplasia Type 1, machine learning, natural language processing, publication analysis, rare diseases

## Abstract

**Introduction:**

This study aimed to investigate the landscape of Multiple Endocrine Neoplasia Type 1 research during the last 22 years using machine learning and text analysis.

**Method:**

In December 2021, all publications indexed under the MeSH term “Multiple Endocrine Neoplasia Type 1” were obtained from PubMed. The whole set of search results was downloaded in XML format, and metadata such as title, abstract, keywords, mesh words, and year of publication were extracted from the original XML files for bibliometric evaluation. The Latent Dirichlet allocation (LDA) topic modeling method was used to analyze specific themes.

**Results:**

This study eventually contained 1,407 publications. Among them, there are 768 (54.58%) case reports and reviews. Text analysis based on MeSH words revealed that the most often studied clinical areas include therapy efficacy, prognosis, and genetic diagnosis. The majority of basic study is focused on genetic alterations. The LDA topic model further identifies three topic clusters include basic research, treatment cluster, and diagnosis cluster. In the basic research cluster, many studies are focused on the expression of Menin. The primary focus of the therapy cluster is pancreatic resections and parathyroidectomy. In the diagnose cluster, the main focus is on Genetic Diagnosis and screening strategies for Hereditary Cancer Syndrome.

**Conclusion:**

The current state of research on MEN1 is far from adequate. Research on rare diseases MEN1 necessitates implementing a broad research program involving multiple centers to advance MEN1 research together.

## Introduction

Multiple Endocrine Neoplasia Type 1 (MEN1) is a rare disease of the endocrine system (1). The incidence of MEN1 is extremely low, but based on the majority of reports available to date, it can be estimated that the incidence of MEN1 is approximately 1–3 per 100,000 but may go undocumented due to the mild clinical presentation of some patients (2). The disease causes a series of endocrine tumors, even malignant ones, at high risk (2, 3). The diagnosis of MEN has been an intractable problem. Until now, there is no consensus on its optimal diagnostic criteria (4). It is likely to go undetected due to mild clinical symptoms, while the diagnosis is more difficult in children and adolescents (4). Therefore, there is a great gap in MEN's clinical diagnosis and treatment, which can negatively affect the prevalence of cancer, and various related clinical issues need to be addressed.

Fortunately, the disease is starting to gain attention in various countries, especially in France and the Netherlands (2). However, the current research on MEN has many loopholes and gaps due to the lack of reasonable control of the research direction. For example, the current MEN diagnosis can be made by genetic testing. However, there are a series of complex ethical issues, so there is a need to find other new diagnostic methods (5). Additionally, the early detection and diagnosis of MEN in children and adolescents is also an urgent issue that needs to be addressed. Some children with the disease are not even diagnosed until later in life (6). Therefore, there is an urgent need to grasp and evaluate the research prospects of MEN in order to solve real clinical problems with the highest efficiency.

There are several software and machine learning methods to analyze the evolving trends in different literature (7, 8). However, one major challenge when using Pubmed for theme analysis is that the PubMed database does not provide topic or concept changes over time (9). Using a semi-automatic approach based on text analysis to identify critical topics is an alternative solution since there is no need to pre-define terms such as MeSH. Therefore, using topic models can better complement the study (9). LDA is the most common approach among the thematic models and has been successfully applied in different fields in similar medical publications research (9–11).

This paper will retrieve and bibliometrically examine MEN1-related articles published and indexed in PubMed during the past 22 years (2000–2021). We will analyze the changes in research directions and findings on MEN1 over the past 22 years and identify the underlying patterns so that researchers can grasp the overall status and research trends related to MEN1 and address a range of relevant clinical issues.

## Method

This study referred to similar previous research methods (10, 11).

### Data Sources and Pre-processing

We downloaded all publications indexed under the Mesh term “Multiple Endocrine Neoplasia Type 1” from PubMed in December 2021. The complete record of the search results was downloaded in XML format, and then the metadata was extracted separately from the original XML file. The metadata extracted in this study included the title, abstract, keywords, Mesh words, and year of publication of each article.

### Topic Modeling

In this study, we use further analysis of abstracts from the raw data to perform LDA modeling analysis to analyze the current macroscopic view of the research field together with MeSH words. LDA is a topic probability model. It can give the probability that an article is relevant to a particular research topic based on how often the term appears in the document set. In LDA, each topic is modeled as an infinite mixture on a set of underlying topic probabilities. In the context of text modeling, topic probabilities provide an explicit representation of the document. By analyzing the abstracts of the articles, the topic with the highest probability is defined as the main topic of each article based on the topic probability calculated by the algorithm.

Analysis using the LDA method generally requires setting the number of themes in advance, which in other previous studies has generally been set to 50 (12, 13). After a preliminary exploration using four different methods (Supplementary Material), we sought to have LDA identify the 20 most characteristic themes.

### Document Clustering

We perform cluster analysis using the Louvain algorithm to build a topic similarity network, which identifies relationships between topics and determines communities of related topics. For each article, we identified the two topics with the highest attribution probability, counted the frequency of each topic in each document, and linked each topic.

All relevant Python and R language codes can be found in the cited literature (10, 11). All descriptive statistics are reported as mean ± standard deviation. The network visualization in the article was performed using Excel and Gephi (https://gephi.org/) (14, 15). This article is a bibliometric analysis and does not require institutional review board or ethics committee approval.

## Results

**Figure 1:**
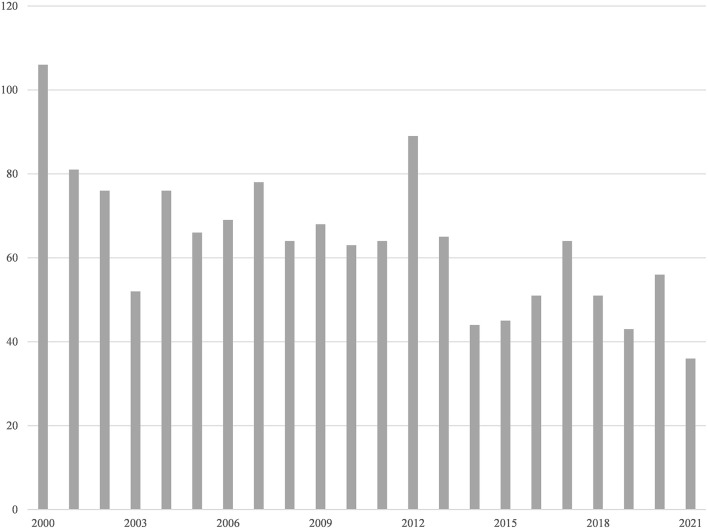
PubMed search results: articles per year.

By searching for characters we identified 1,407 publications, including 406 Case Reports, 362 Reviews, and 19 Clinical Trials. As can be seen, there is a trend of relative decrease in the number of publications from 2000 to the present (Figure 1), with an average of 50 studies related to MEN1 type published in the last 5 years.

### MeSH Analysis

**Figure 2:**
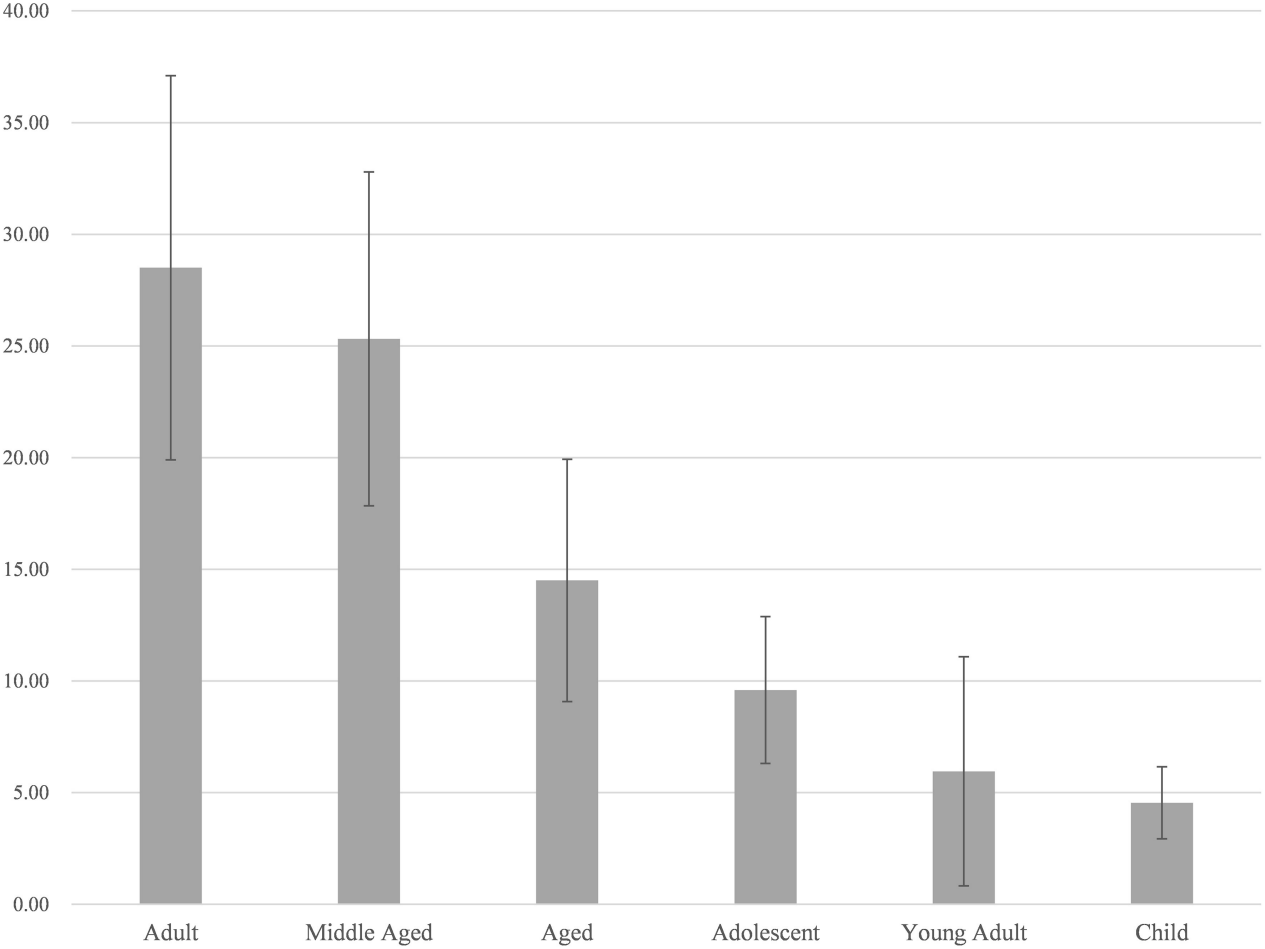
Annual output of literature, broken down by age group.

After removing disease-specific words such as Multiple Endocrine Neoplasia Type 1 and Humans words from the MeSH terminology, the MeSH words were further analyzed. Table 1 shows the top 20 Mesh terms that appear in the retrieved articles that are related to the topic. The most frequently occurring terms include Pancreatic Neoplasms, Proto-Oncogene Proteins, Mutation, and others. Figure 2 further shows the percentage of the number of relevant research publications for different age groups in the last 22 years. It is important to emphasize that articles dealing with more than one age group can be in the total number of publications for all corresponding age groups. Overall, Adult and Middle Aged were the most prominent in the literature covered in this study, followed by Aged, with child coming in last.

**Table 1:** Overall ranking of research foci in the past 22 years.

**Rank**	**MeSH term**	**Record of occurrence in publications**
1	Pancreatic neoplasms	477
2	Proto-oncogene proteins	353
3	Mutation	221
4	Neuroendocrine tumors	196
5	Pituitary neoplasms	165
6	Adenoma	163
7	Parathyroid neoplasms	159
8	Hyperparathyroidism	152
9	Gastrinoma	147
10	Germ-line mutation	141
11	Insulinoma	136
12	Retrospective studies	136
13	Animals	133
14	Pedigree	121
15	Hyperparathyroidism, Primary	115
16	Treatment outcome	104
17	Parathyroidectomy	103
18	DNA mutational analysis	98
19	Tomography, X-ray computed	98
20	Zollinger-Ellison syndrome	97

Considering that MEN1 is a rare disease and the overall number of studies is low, and the number of publications per year is relatively decreasing. Therefore, instead of looking for fast-growing domains, we show here the results for a larger number of MeSH words. We divided it into two aspects, the results of MeSH words related to treatment and the results of MeSH words related to basic research.

**Figure 3:**
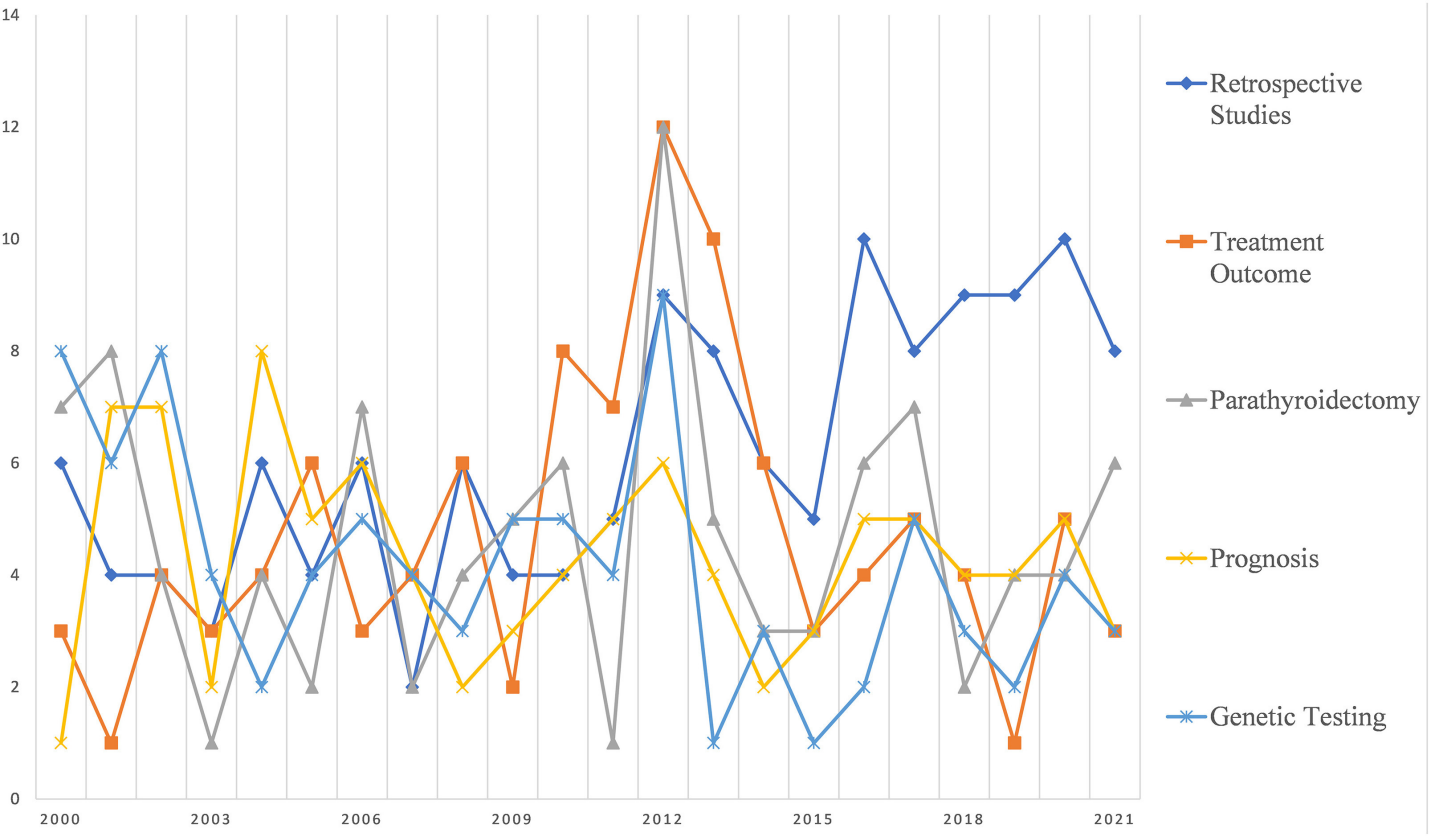
Research foci trends related to clinical research.

Figure 3 shows the trends in the five topics with the highest number of clinical studies. First of all, it can be seen that retrospective studies make up the majority of the MEN1 type. In addition, treatment efficacy and prognosis are also the focus of attention in MEN1 clinical studies. Genetic diagnosis is also a very important part for MEN1. It is interesting to note that although MEN1 is a multiple neoplastic disease, there is a large body of literature on parathyroidectomy that focuses on this topic.

**Figure 4:**
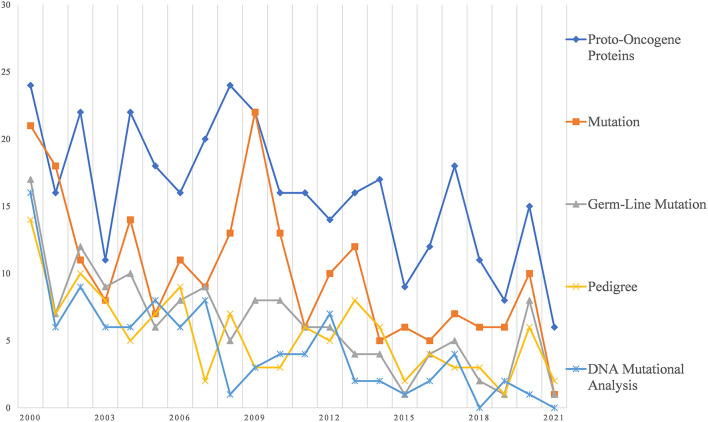
Research foci trends related to basic research.

Figure 4 further illustrates the research hotspots related to basic research. It can be seen that most of the words related to basic research are related to gene mutation and analysis. The most frequent occurrence was Proto-Oncogene Proteins. The second in line is Mutation, where Germ-line Mutation is the focus of Mutation. Pedigree and DNA Mutational Analysis were also the focus of the study.

### Latent Dirichlet Allocation

**Figure 5:**
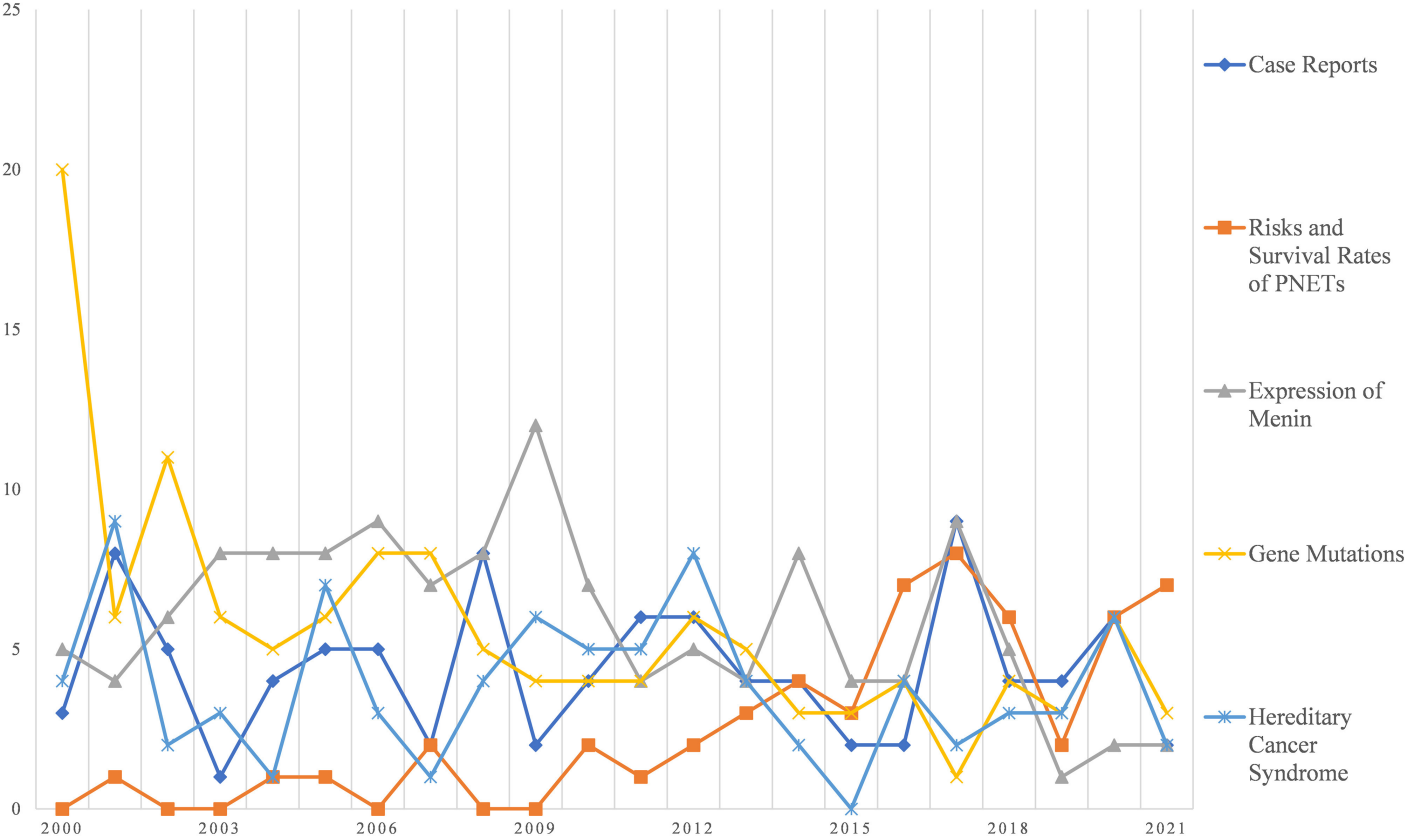
Latent Dirichlet allocation (LDA) analysis: top 5 topic areas in late 22 years.

Machine learning algorithms were used to give more specific details to the study topics, and a total of 20 topics in MEN1 were identified in this study. Figure 5 shows the trend of the five most numerous themes over the last 11 years. It can be seen that *Case Reports* is the most studied topic in MEN1. PENTs have also received increasing attention in recent years. In addition, *Expression of Menin, Gene Mutation*, and *Hereditary Cancer Syndrome* have also received a lot of attention. It is noteworthy that in the field of MEN1, there were relatively more studies on *Gene Mutations* in the early years, while declining in recent years.

**Figure 6:**
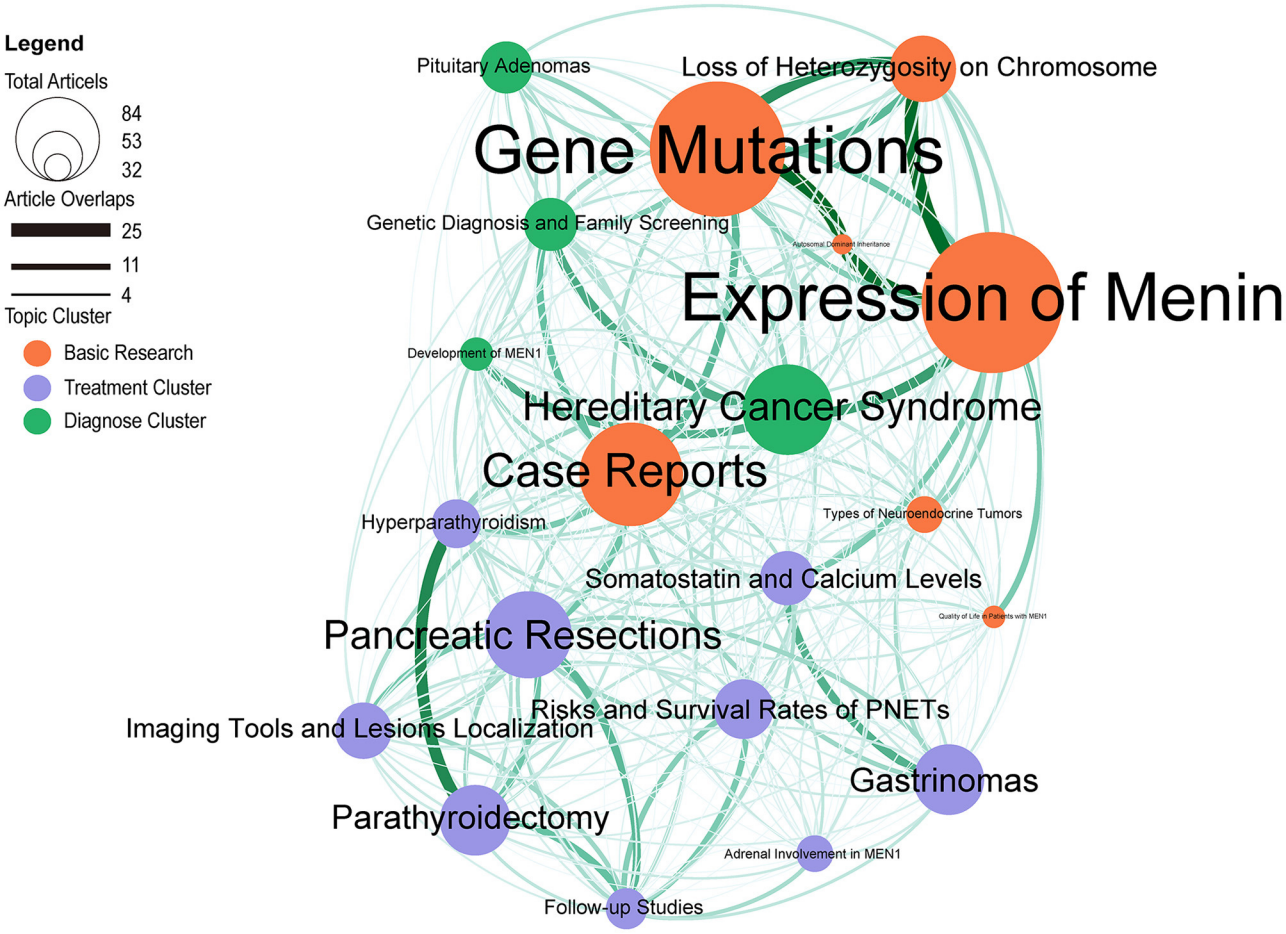
LDA research topic cluster network: inter-and intra-relationships.

Network analysis of topics can present clusters of topics with high similarity, and we can study network analysis of topics to aggregate domains of similar topic clusters and show the connection and strength between different topics. We identified three thematic network clusters by the Louvain method (Figure 6), with strong intra-cluster relationships among publications within each thematic network cluster. These 3 thematic network clusters can be grouped into 3 directions in the MEN1 research area: Diagnosis, Treatment, and Basic Research. For each topic network, the size of the bubble represents the number of papers it is associated with. The lines between the bubbles indicate that the two topics contain articles that use common terms, and their thickness represents the magnitude of the relationship between the topics.

In the Diagnosis cluster, the most studied topics are *Hereditary Cancer Syndrome, Genetic Diagnosis and Family Screening, Pituitary Adenomas*, and *Development of MEN1*. Among them, *Genetic Diagnosis and Family Screening* is closely related to *Hereditary Cancer Syndrome* and *Case Reports*. In addition, *Hereditary Cancer Syndrome* showed the connection with *Development of MEN1* and *Expression of Menin*.

In the Basic Research cluster, the most studied topics are *Expression of Menin* and *Gene Mutations*. It can be seen that *Expression of Menin* and *Gene Mutations* both show more connections with *Loss of Heterozygosity on Chromosome* and *Autosomal Dominant Inheritance*. Interestingly, we can notice that *Case Reports* was recognized by machine learning and entered other words related to basic research. The possible reason is that the basic research for these cases is mostly based on rare cases.

In the Treatment cluster, you can see that the hot spots are concentrated in *Pancreatic Resections* and *Parathyroidectomy*. Within this cluster, we see that *Parathyroidectomy* is most closely related to *Hyperparathyroidism*. *Anti-TNF*α *Agents* are clearly related to *Follow-up Studies, Clinical Response and Remission* and *Combination Therapy*. *Imaging Tools and Lesions Localization* and *Follow-up Studies* have extensive connections with other topics in the cluster. This suggests that accurate location of lesions is a research hotspot in treatment, and *Follow-up Studies* is the main research form.

## Discussion

From 1903, when the first case of MEN1 was described, to 2012, when the latest clinical guidelines were updated, many research efforts have led to breakthroughs that have improved our understanding of MEN1 (2). The MEN1 locus was first clearly localized to 11q13 in 1988 (16). The identification of MEN1 and its protein product MENIN in 1997 further paved the way for the diagnosis of the gene (17, 18). Our analysis of the literature since 2000 shows that the subsequent two decades have been closely focused on these themes, such as the study of further mechanisms of these genes and their products, genetic diagnosis and family screening strategies. However, these studies are still not clearly elucidated, for instance, the imaging of survival rates by genetic monitoring still needs further elucidation (2). So these existing research hotspots will remain an area for further research in the future. Furthermore, some areas that have been explored but are still insufficient were also identified in this study, such as the study of quality of life in MEN1 patients still needs further elucidation in future studies.

In our study, we found that research on PENT has received increasing attention in recent years. Duodenopancreatic ENTs are the first manifestation in approximately 20% of MEN1 patients (19). The previous diagnosis of duodenopancreatic ENTs is usually a mass syndrome or found during systematic imaging, which usually accounts for about 55–70% of patients (20–22). But microscopic tumors were able to be detected in 95% of the patients (6). Thus, the presence of duodenopancreatic ENTs may be underestimated. As it is one of the leading causes of death in men (19), one of the major breakthrough directions for further research will be how to examine the mechanistic relationship of duodenopancreatic ENTs in MEN1.

Rare diseases are now considered to be a global public health problem (23). With the support of supervision and economic incentives, researchers have made a lot of efforts to promote the development of treatments for rare diseases (24). In fact, some studies have pointed out that the funds received by companies that actually focus on RDS have increased, while the financial support for common diseases has decreased (25). However, no rapid increase in the number of research articles has been observed in the MEN1 field as compared to other fields. The reasons for this may be multifaceted. The first reason may be the underdiagnosis of MEN1, for which some patients are actually underdiagnosed. In addition due to the lack of rare cases, greater barriers to the development and availability of drugs to treat these diseases may be one of the reasons.

Rare case reports and reviews accounted for about half of the published articles in this study. This may be due to the small incidence, so a large number of studies are presented in the form of cases. Additionally, a significant number of reviews were included in this study, possibly in conjunction with references to MEN1 in the context of rare diseases. In any case, publications on rare diseases demonstrate that landscape is significantly different from other diseases, and this suggests a need for systematic change in the study of rare diseases in order to advance the research on MEN1 more. Novel evolutionary and sequencing technologies have progressively revealed mutated genes in MEN1, but the translation of these into therapeutics has lagged far behind the rate of production of this knowledge (23). For MEN1, the selection of the optimal drug therapy is challenging for patients because clinical trials are usually not conducted in MEN1 patients, but rather extrapolate results from trials conducted in patients with a single endocrine tumor or based on descriptive small cohort studies (26). How to establish a more standardized treatment process and allow large centers to collect enough case information and pathological specimens for research will be one of the future research directions.

There are several limitations to this study. First, there are other databases available for bibliometric research besides PubMed, including the Web of Science, Scopus, and Embase. It is worth noting, however, that PubMed contains the highest quality peer-reviewed research and excludes irrelevant, non-peer-reviewed publications. If future researchers are able to explore publications in other medical science databases, they will be able to provide a more comprehensive and detailed description of the field than we have provided here. In addition, some recently published papers may not appear in our study because they have not been indexed by MeSH words. These are shared limitations of published studies. Finally, the LDA themes and their connections in this study were created by artificial intelligence, which presents a machine-driven understanding. A deeper, more detailed exploration of these topics can provide easier interpretation and more accurate results, so medical professionals can provide more effective treatment.

## Conclusions

The current state of research on MEN1 is far from adequate. Current clinical research on MEN focuses on therapy efficacy, prognosis, and genetic diagnosis, while basic research focuses on genetic alterations and expression of Menin. Research on MEN1 necessitates implementing a broad research program involving multiple centers to advance research together.

## Data Availability Statement

The raw data supporting the conclusions of this article will be made available by the authors, without undue reservation.

## Author Contributions

YF and SC: conceptualization, supervision, and writing—review and editing. CF and HC: methodology. CF, LH, and HC: formal analysis. CF: investigation. CF and LH: writing—original draft preparation. All authors contributed to the article and approved the submitted version.

## Funding

This work was supported by the Hunan Provincial Natural Science Foundation of China (Grant No. 2021JJ30929) and the National Natural Science Foundation of China (Grant No. 81802620).

## Conflict of Interest

The authors declare that the research was conducted in the absence of any commercial or financial relationships that could be construed as a potential conflict of interest.

## Publisher's Note

All claims expressed in this article are solely those of the authors and do not necessarily represent those of their affiliated organizations, or those of the publisher, the editors and the reviewers. Any product that may be evaluated in this article, or claim that may be made by its manufacturer, is not guaranteed or endorsed by the publisher.

## References

[1] Hoff AO Cote GJ Gagel RF. Multiple endocrine neoplasias. Annu Rev Physiol. (2000) 62:377–411. 10.1146/annurev.physiol.62.1.377 10845096

[2] Al-Salameh A Cadiot G Calender A Goudet P Chanson P. Clinical aspects of multiple endocrine neoplasia type 1. Nat Rev Endocrinol. (2021) 17:207–24. 10.1038/s41574-021-00468-3 33564173

[3] Dreijerink KM Goudet P Burgess JR Valk GD. Breast-cancer predisposition in multiple endocrine neoplasia type 1. N Engl J Med. (2014) 371:583–4. 10.1056/NEJMc1406028 25099597 PMC4243053

[4] Lodish MB. Careful investigation of a rare disease: insights into multiple endocrine neoplasia type 2B. Lancet Diabetes Endocrinol. (2019) 7:167–8. 10.1016/S2213-8587(18)30353-X 30660596

[5] Lips CJ Höppener JW. Ethics: Genetic testing for MEN1–whose responsibility? Nat Rev Endocrinol. (2012) 8:575–6. 10.1038/nrendo.2012.164 22965167

[6] Yates CJ Newey PJ Thakker RV. Challenges and controversies in management of pancreatic neuroendocrine tumours in patients with MEN1. Lancet Diabetes Endocrinol. (2015) 3:895–905. 10.1016/S2213-8587(15)00043-1 26165399

[7] Névéol A Zweigenbaum P. Clinical natural language processing in 2015: leveraging the variety of texts of clinical interest. Yearb Med Inform. (2016) 10:234–9. 10.15265/IY-2016-049 27830256 PMC5171575

[8] Chen X Xie H Wang FL Liu Z Xu J Hao T. A bibliometric analysis of natural language processing in medical research. BMC Med Inform Decis Mak. (2018) 18:14. 10.1186/s12911-018-0594-x 29589569 PMC5872501

[9] Gal D Thijs B Glänzel W Sipido KR. Hot topics and trends in cardiovascular research. Eur Heart J. (2019) 40:2363–74. 10.1093/eurheartj/ehz282 31162536 PMC6642725

[10] Stout NL Alfano CM Belter CW Nitkin R Cernich A Lohmann Siegel K. A bibliometric analysis of the landscape of cancer rehabilitation research (1992-2016). J Natl Cancer Inst. (2018) 110:815–24. 10.1093/jnci/djy108 29982543 PMC6279275

[11] Feng C Wu Y Gao L Guo X Wang Z Xing B. Publication landscape analysis on gliomas: how much has been done in the past 25 years? Front Oncol. (2019) 9:1463. 10.3389/fonc.2019.01463 32038995 PMC6988829

[12] Blei DM Probabilistic topic models. Commun ACM. (2012) 55:77–84. 10.1145/2133806.2133826

[13] Blei DM Ng AY Jordan MI. Latent dirichlet allocation. J Mach Learn Res. (2003) 3:993–1022. 10.5555/944919.944937

[14] Jacomy M Venturini T Heymann S Bastian M. ForceAtlas2, a continuous graph layout algorithm for handy network visualization designed for the Gephi software. PLoS ONE. (2014) 9:e98679. 10.1371/journal.pone.0098679 24914678 PMC4051631

[15] Bastian M Heymann S Jacomy M. Gephi: an open source software for exploring and manipulating networks. In: Third international AAAI Conference on Weblogs and Social Media. San Jose (2009).

[16] Larsson C Skogseid B Oberg K Nakamura Y Nordenskjöld M. Multiple endocrine neoplasia type 1 gene maps to chromosome 11 and is lost in insulinoma. Nature. (1988) 332:85–7. 10.1038/332085a0 2894610

[17] Chandrasekharappa SC Guru SC Manickam P Olufemi SE Collins FS Emmert-Buck MR. Positional cloning of the gene for multiple endocrine neoplasia-type 1. Science. (1997) 276:404–7. 10.1126/science.276.5311.404 9103196

[18] Lemmens I Van de Ven WJ Kas K Zhang CX Giraud S Wautot V. Identification of the multiple endocrine neoplasia type 1 (MEN1) gene. The European Consortium on MEN1. Hum Mol Genet. (1997) 6:1177–83. 9215690 10.1093/hmg/6.7.1177

[19] Goudet P Murat A Binquet C Cardot-Bauters C Costa A Ruszniewski P. Risk factors and causes of death in MEN1 disease. A GTE (Groupe d'Etude des Tumeurs Endocrines) cohort study among 758 patients. World J Surg. (2010) 34:249–55. 10.1007/s00268-009-0290-1 19949948

[20] Donegan D Singh Ospina N Rodriguez-Gutierrez R Al-Hilli Z Thompson GB Clarke BL. Long-term outcomes in patients with multiple endocrine neoplasia type 1 and pancreaticoduodenal neuroendocrine tumours. Clin Endocrinol. (2017) 86:199–206. 10.1111/cen.13264 27770475

[21] van Asselt SJ Brouwers AH van Dullemen HM van der Jagt EJ Bongaerts AH Kema IP. EUS is superior for detection of pancreatic lesions compared with standard imaging in patients with multiple endocrine neoplasia type 1. Gastrointest Endosc. (2015) 81:159–67.e2. 10.1016/j.gie.2014.09.037 25527055

[22] Barbe C Murat A Dupas B Ruszniewski P Tabarin A Vullierme MP. Magnetic resonance imaging versus endoscopic ultrasonography for the detection of pancreatic tumours in multiple endocrine neoplasia type 1. Dig Liver Dis. (2012) 44:228–34. 10.1016/j.dld.2011.09.014 22078814

[23] Tambuyzer E Vandendriessche B Austin CP Brooks PJ Larsson K Miller Needleman KI. Therapies for rare diseases: therapeutic modalities, progress and challenges ahead. Nat Rev Drug Discov. (2020) 19:93–111. 10.1038/s41573-019-0049-9 31836861

[24] Tambuyzer E. Rare diseases, orphan drugs and their regulation: questions and misconceptions. Nat Rev Drug Discov. (2010) 9:921–9. 10.1038/nrd3275 21060315

[25] Roessler HI Knoers N van Haelst MM van Haaften G. Drug repurposing for rare diseases. Trends Pharmacol Sci. (2021) 42:255–67. 10.1016/j.tips.2021.01.003 33563480

[26] Brandi ML Agarwal SK Perrier ND Lines KE Valk GD Thakker RV. Multiple endocrine neoplasia type 1: latest insights. Endocr Rev. (2021) 42:133–70. 10.1210/endrev/bnaa031 33249439 PMC7958143

